# Supplementary figures and images for: Rehabilitation outcomes of bird-building collision victims in the Northeastern United States
Source: PLoS One. 2024 Aug 7;19(8):e0306362. doi: 10.1371/journal.pone.0306362 (PMC11305546; doi:10.1371/journal.pone.0306362)

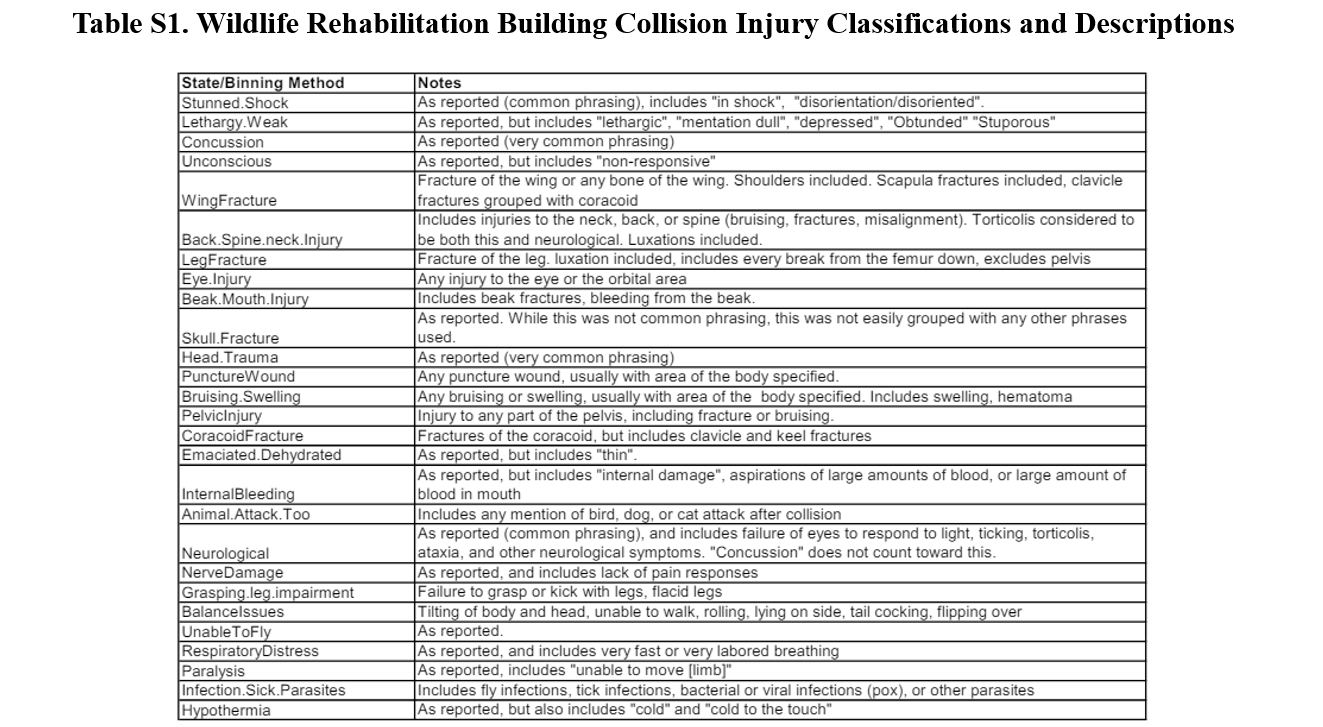

Supplement: S1 Table — Table listing the injury classifications that were used, and notes on injury descriptions and classification rules aiding in binning cases by injury similarity despite highly varied language or word choice. (PNG) [file pone.0306362.s002.png]
