# Supplementary material for: Rehabilitation outcomes of bird-building collision victims in the Northeastern United States
Source: PLoS One. 2024 Aug 7;19(8):e0306362. doi: 10.1371/journal.pone.0306362 (PMC11305546; doi:10.1371/journal.pone.0306362)
Supplement: Attachment — Submitted filename: Review _ Rehabilitation Outcomes.docx [file pone.0306362.s003.docx]

This paper addresses a basic but significant gap in the literature – the fate of birds that hit glass but do not die immediately. To grasp the magnitude of mortality from this source, it is essential to learn as much as possible about this group of birds. The authors have worked hard to extract useful information from a very difficult but important data set. The language is clear throughout as is the logic of the analysis. They have done an excellent, and comprehensive review of the relevant literature, including papers that are still in review. There are some relatively minor copy editing issues. I had some difficulty accessing the data spreadsheets.

Specific comments:

L 20 Klem, 1990 and 2024 do suggest die at some rate, so possibly say ‘largely assumed to survive’; similarly line 44 and line 73

L 95/125 – are better data available directly from rehab centers, as opposed to state reports?

L143 – individual centers may also be a source of variation – are data sources identifiable? (they must be, since funding is a variable)

L150 – what does ‘complex nature of reporting’ mean?

L156 what was rationale for expecting population density to have an impact?

L160 – why might you expect that organizational funding level would have an impact?

L215 why not use data from all counties in NY? How many were not selected?

L230 why was this data not available?

Table 2 – state that families are in order of %release

Seems as though head trauma, concussion and balance issues may not be independent

L280 + % released not meaningful to two decimal places

L293 ‘vast majority …. appeared’ – how do you know? Were all examined for parasites?

L328 ‘no effect …… or … funding’ -- but line 311 indicates no effect of funding, also

L346 It is possible that collisions behavior is quite different at night, with birds not flying directly at glass, but coming down nearby and fluttering towards it – may not be able to generalize from this paper.

L365 why would younger birds require protracted care? They have fledged and are migrating

L366 you would expect that fractures would take longer to resolve than head trauma; rehabilitators may also be dealing with many more cases in summer; may retain birds rather than release in winter, if weather is bad

L377 Territorial attacks on reflections should not be considered with collisions – they generally do not cause injuries, although the birds may waste significant energy

L400-401 Chi square and Fishers? Not mentioned in analysis section

L414 Well funded orgs may not be able to handle more cases, although they might have better equipment, facilities and access to vet care. How many orgs were involved? Some very poorly funded orgs have very passionate and dedicated rehabilitators

L420-423 duplicated in L423-446

L442 ‘representative’?

L447 starting to be MOTUS data on survival of rehab releases

L449 Given that birds may be transported significant distances to rehab facilities, how will more fine grained maps of population density improve the analysis?
