# Supplementary material for: Rehabilitation outcomes of bird-building collision victims in the Northeastern United States
Source: PLoS One. 2024 Aug 7;19(8):e0306362. doi: 10.1371/journal.pone.0306362 (PMC11305546; doi:10.1371/journal.pone.0306362)
Supplement: Attachment — Submitted filename: Review Responses _ Rehabilitation Outcomes.docx [file pone.0306362.s004.docx]

**Response to Reviewers**

Dear Dr. Pukenis Tubelis,

We appreciate the valuable insights and feedback you and the reviewers provided on our manuscript. Please see below for a detailed response to each comment.

Sincerely,

Ar Kornreich, Dustin Partridge, Mason Youngblood, and Kaitlyn Parkins

Additional Editor Comments:

Please find my review below:

Introduction
Very well written, as occurred with the objectives.
Line 39. Do not indent the first paragraph of a given section. Only the next ones.
Line 46. You have to change to [8 ̶10]. When you have 3 or more refs in immediate sequence, you have to use a long dash (not hyphen) between the initial and final numbers. Please check this throughout.
Line 48/49. It has to be [11–14].
Line 50. “12” should be the first number within the brackets. Keep always an increasing order.
Line 58. Change to [6, 21–25]. Please follow this format along the manuscript.

**These corrections have been made in the manuscript.**

Line 71. I think you have to delete “´s 2022 study”. Just keep the author and the brackets with number.

**This has been fixed in the manuscript.**

Line 129. You could add birds and US to the table title.

**This has been fixed in the manuscript.**

Line 158. Delete the space between the second bracket and the comma. Delete the comma and space after “location”.

**This has been fixed in the manuscript.**

Line 165. I think you have to delete “´s 2014 study”.

**This has been fixed in the manuscript.**

Line 244. It is better to change to “varied”. Consider always using past in the Results.

**This has been fixed in the manuscript.**

Line 262. Fix the brackets in italics.

**This has been fixed in the manuscript.**

Lines 264 to 265. As these numbers after families (n = xx) are found in the Table, there is no need to repeat them here. I suggest deleting them from the text.

**This has been fixed in the manuscript.**

Line 270. I think that the figure caption has to be more informative (birds, n states, US…).

**This has been fixed in the manuscript.**

Line 274. Its title is not much informative. I suggest the same for the figure caption.

**This has been fixed in the manuscript.**

Why are the names in italics ? (first column and first line of the table).

**This has been fixed in the manuscript.**

Line 313. Table 3. Please check if I´m wright or wrong….should you add something like “Variables” in the cell above that of Mass?
**Yes, we have added “Variables” in the cells above “Mass”.**

Line 331. Table 4. Explain the bold in some cells. Or change it.

**We have removed it, as it is redundant with the asterisks marking the significant effects.**

Discussion
I suggest you add Sut-titles for sections of the Discussion. It eases the reding and the finding of specific parts. Not mandatory, but very welcome.

**We have added subtitles to the Discussion section.**

Line 255. I think you have to delete “2022”.

**This has been fixed in the manuscript.**

Lines 423-425. It is a too short paragraph. Can you merge it with other ?

**We have expanded on this paragraph in the manuscript to highlight the labor of wildlife rehabbers and the explanatory effect it may have on our findings.**

Lines 413-438. Ops! Here you have a full page (19) without the citation of references/papers. Can you please revise this section, and try to add some studies to compare and or support your thoughts/findings. Ah! Now I saw that this problem extends to line 469. Please try to improve this final part of the Discussion. It is too “clean”. Consider an international audience.

**We have added citations and more context regarding seasons to increase accessibility with an international audience.**

References
Page numbers of articles and chapters have to be separate by a long dash and not hyphen.
You have to add the DOI for all references that have it (mostly articles). Use the format <https://doi.org/numbers> and letters. Check recent issues if in doubt.
**This has been corrected, thank you!**

Supplementary Material
Along the text, you used something like “see Supplementary Materials”. You should be more specific, like mentioning the table, figure, etc… Please check the instructions again. Still about this, I could not find the links for them in the pdf that I have to evaluate your submission. I do not know if you provided them or not during the submission. Please check this when you submit the corrected version ok? They will be necessary. Maybe it was not your fault but a problem in the system…

**Our deepest apologies for this. The Supplementary Materials were and are accessible through our Data and Code Availability Statement. This has been made clearer in the manuscript.**

**5. Review Comments to the Author**

Please use the space provided to explain your answers to the questions above. You may also include additional comments for the author, including concerns about dual publication, research ethics, or publication ethics. (Please upload your review as an attachment if it exceeds 20,000 characters)

Reviewer #1: I consider the results presented in the manuscript to be very beneficial for a better view of the threats to bird species in nature, especially because some of them are still underestimated. The work is clearly written, the literature review and the discussion is of high quality, however, for a better understanding, I suggest some explanation, especially in methodology.

Line 87 - You state that wildlife rehabilitators are licensed by their state - what the conditions are for obtaining a license? I suppose it varies from state to state. Are at least some of the conditions for issuing a license the same or similar, for example, do the conditions for obtaining this license contain certain knowledge (e.g. factors affecting the survival of animals in nature, the basics of treatment, etc.) and their verification, for example by an exam?

**We agree that these are all very good questions, but we are unable to address these here because they are out of the scope of this manuscript. A policy review of state policies for granting licenses would be a welcomed addition to the literature and could lead to more cohesive guidelines.**

Line 244 – Why do some seasons include three months and others only two? I assume that in your latitudes the conditions are different from ours, where the year would be divided into four seasons of three months each. On what basis was the division chosen? Can such a division not affect the results of the number of individuals admitted for the given period?
**In the global north, season divisions are based on Loss et al. 2014, based on migration and breeding time-frames. More descriptive detail will be added to the manuscript. Because of the seasonal nature of the study area, and the differences in collisions expected between seasons that are regularly studied in the northeast, we believe it is important to divide by biological seasonality rather than by equal numbers of months.**

281 – 292 It is not clear how the individual types of injuries differ from each other - for example, how does a concussion differ from a balance issue, as I assume that even with a concussion, balance problems can occur. I would suggest listing the individual categories in the methodology with an example so that it is clear how they differ from each other. Likewise, information on diagnosis is missing - is a veterinarian present and are any special examinations (for example, X-rays) carried out?

**Unfortunately, we are bound by the level of detail in the report. Oftentimes, the report simply states injuries as “Head Trauma”, “Concussion”, “Unable to Fly”, etc. With the exception of data from Tri-State Bird Rescue and Research and other very rare exceptions in records from Pennsylvania, in-depth veterinary records were not available to us.**

Line 436 - Is it typical for these rehabilitation centres that they care for only one group of animals (e.g. birds) or are all animals admitted to the rehabilitation centres (including mammals, etc.)? This may also reflect the specialization of these centres and thus better knowledge of the given species.
**This varies, but only the minority specialize in only avian species, and most organizations will take in small mammals and/or reptiles as well. There are some rehabbers that primarily or only accept bird-of-prey or songbird species, but the majority or organizations accept most native terrestrial vertebrates.**

—-------------------------------------------------------------------

Reviewer #2: Major comments:
In this study, the authors collate a large data set and explore predictors of bird-building collisions throughout the Northeastern United States. The taxonomic scope of the data set is impressive, spanning some 152 spp., and the authors report significant effects of season and body mass, among others, on aspects of bird-building collisions.
Overall, I thought that the study has merit and could be of interest to a wide audience. However, in its current form I feel that it requires major revision before it can be deemed suitable for publication.
Specifically, as a reviewer I often found myself confused. This often indicates that extra clarification is needed to help the reader fully understand what the authors have done and more importantly, why? This is particularly the case surrounding the statistics employed here. There were some statements which I disagreed with, and I have highlighted these in my list of minor comments below. I encourage the authors to read and cite more widely within the wildlife rehabilitation literature – there are many studies that should be acknowledged here.
Importantly, I was left wondering what the a priori expectations of testing age, body mass, funding, etc., would be given previous knowledge on bird-building collisions. At the end of the introduction, I would expect to find the authors’ expectations evidenced by published research. Inclusion of this, and discussion of each point in sequential order in the Discussion would largely improve the paper.
Given the nature of this study and the findings, I also expect the Discussion section to be full of evidence-informed management actions to prevent bird-building collisions in the future. Currently, there are none. What about decals on windows, habitat management, tinting glass, etc.? This seems like an obvious oversight by the authors and I am sure they will welcome the opportunity to apply their findings in such a way.
As previously mentioned, I believe this study has merit and I hope that the authors receive my comments in a constructive manner, in which they were intended.
Please see point-by-point comments below.

**Thank you for your constructive feedback. We have added points to help clarify our intent, added more wildlife rehabilitation literature, and, importantly, added recommendations for how to prevent collisions based on existing literature.**

Minor comments:
Title/abstract – the title refers to “building collisions” but “window collisions” are used throughout the abstract. I would suggest being consistent in use of terminology.

**This has been fixed in the manuscript.**

Abstract
Line 19 – replace second “birds” with “those” (the word birds is included three times in one sentence here).

**This has been fixed in the manuscript.**

Lines 22-24 – there seems to be a sentence missing in your summarised methods here. For example, you state that patterns of survival and release were examined but the next sentence begins with seasonal differences. I suggest adding something along the lines of:
“In addition to this, we examined temporal trends in bird-window collisions and compared differences between species across seasons…”.

**Added a note in this sentence to acknowledge we looked at trends across multiple seasons.**

Lines 24-26 – split into two separate sentences, it will read better.

**This has been fixed in the manuscript.**

Lines 26-27 – this seems a bit obvious, consider omitting to save on word count.

**Thank you for the suggestion but while rehabilitators may expect that concussion and head trauma are the most common injury, it is still a result of our study and may be less obvious to readers not as familiar with building collisions.**

Lines 30-31 – this seems to contradict the statement on lines 20-21.

**This is precisely one of the reasons we find this study was valuable; it challenges the held assumption that once a patient survives the initial collision, their prognosis is largely going to be fine. Our data suggests that collision victims face serious risk of death for at least 48 hours after the collision occurs.**

Line 32 – “This study reports different data than carcass studies” most of the rehab literature is based mainly on live individuals admitted to rehab centres, with a strong bias towards individuals that are still alive. I am not quite sure what “carcass studies” the authors allude to here.

**The majority of field-based studies examining the scale of the impact of building collisions are conducted by examining the number of carcasses found by collision monitors (e.g., Loss et al 2014 & 2015, Parkins et al. 2015, Lao et al 2022). We expand upon this in the introduction and appreciate the comment because it highlights the need for increased collaboration between those in field-based conservation and rehabilitation. We were not aware of many of the valuable rehabilitation papers on this topic until beginning this paper and had solely focused on carcass studies.**

Line 36 – what do the authors mean here by “conservation crisis”?

**This has been changed to “this leading threat to wild birds.”**

Introduction
Line 39 – reconsider how the opening sentence begins, as at the moment it is a little difficult to read. What about “Many bird populations throughout North America are declining…”.

**This has been fixed in the manuscript.**

Line 43 – could the authors please expand, perhaps with an example, of how a building can be built to be “bird friendly”?

**This is an important suggestion for protecting birds and we have added this to the conclusion.**

Line 48 – research from the UK has shown a positive relationship between bird-building collisions and increased urbanisation, see: <https://doi.org/10.1002/ece3.8856>

**Thank you for this! That study is now cited in that portion of the manuscript, as citation 11.**

Line 50 – reconsider the use of “work against birds” here.

**This has been changed “work against” to “endanger”**

Line 64 – insert a comma after “scale”.

**This has been fixed in the manuscript.**

Lines 68-69 – the authors claim that most studies use carcass recoveries but only cite a single study here, please add more examples to emphasise the point.

**These have been added to the manuscript.**

Line 71 – replace “collider” with a more suitable term, here. Either “birds” or “victims” will do.

**This has been fixed in the manuscript.**

Lines 71-72 – this sentence could do with some revision, perhaps split into two and emphasise the point that only 7.2% of birds that collided died.

**This has been fixed in the manuscript.**

Line 73 – reconsider the word “sharp” here.

**This has been changed to “drastic” in the manuscript.**

Line 74 – replace “to” with “and”.
**This has been fixed in the manuscript.**

Lines 90-93 – “, as peer-reviewed articles describing or 93 incorporating rehabilitation data are uncommon [51]” this is simply untrue. There are hundreds of peer-reviewed journal articles that present or use rehabilitation data to answer ecological or conservation research questions. A quick search on Google Scholar will return many, many studies
**This has been fixed in the manuscript– we have removed this line.**

Lines 93-95 – again I’m not sure if I agree with this either. The WILD-ONe rehabilitation database is often used in publications. There are also a number of papers published in elsewhere that summarise data from large, collated databases

**Thank you for alerting us to this resource, we have added citations including the usage of these databases, but we still feel as though rehabilitation-based resources are underutilized in conservation research, and that communication between rehabilitators, conservation scientists, and other stakeholders could be improved.**

Line 101 – project?

**This has been changed to “study” in the manuscript**

Lines 107-109 – this is very vague, what do the authors mean by “parts of the avian community”? Please clarify.

**This has been changed to “different bird species” in the manuscript.**

Lines 112-113 – I think the authors need to clarify that they are only collecting data from birds that collided with buildings and were admitted to rehabilitation centres. At the moment, this sentence seems to suggest that they will also account for birds that die later on in the wild.

**We have added some context throughout but feel that our introduction demonstrates that we are studying rehabilitation outcomes. However, your point about survival in the field is important, and one we try to address later on. We assume that birds being brought to rehabilitation is a "best case scenario" for survival. Birds that remain in the wild or on a city street stunned after a collision likely survive far less than 40% of the time.**

Line 114 – “which theoretically improves their chance at survival.” Be careful with the claims here. The authors do not empirically test survival rates between birds admitted to rehab centres with those that were not admitted, so I don’t think they can make this claim.

**We agree it is important not to make claims here but there are several reasons we think this is important to propose, with the caveat "theoretically" included. Most importantly, people or organizations that find a collision victim bring birds to rehabilitators on the assumption that they are more likely to survive with medical assistance. This assumption may be true, if only because stunned birds are easy prey for predators and there are documented cases of gulls, corvids, and raptors hunting stunned birds in front of dangerous glass facades.**

Methods
Line 140 – avoid colloquial language throughout, e.g., “there’s”.

**This has been fixed in the manuscript.**

Line 145 – “or strong suspicion thereof” what does this mean? This seems very subjective, and I am wondering whether I would be able to follow the authors’ methodology perfectly using that phrase.

**This means that entries where the writing rehabber expresses palpable suspicion that building collision is the cause for admission. Wording such as “suspected window strike,” “probably hit window”, or similar were included in the dataset. The manuscript has been revised to be clearer.**

Line 146-147 – this sentence can be removed because it has already been made clear that only bird-building collisions records were extracted.

**Respectfully, we disagree: for future replication of this study, we think this point is important to include because it makes clear that circumstantial evidence of a collision was not enough in and of itself for inclusion in this study.**

Lines 147-148 – what did the authors do with records that had the disposition as “unknown”? It seems important to me that these are not excluded, but are presented descriptively.

**Records that had unknown, “transferred”, or “pending” dispositions were excluded from analyses, as early on the focus of the study was heavily focused on the final outcomes of patients and their treatment time. Obtaining the data for this paper required us to (mostly) manually review 9,497 pages of records with 3 to 30 cases per page. This effort alone took several months. To return to the obtained records in search of omitted entries for a qualitative description is unfortunately not feasible.**

Lines 149-150 – what does this mean? Data were left out because they were too confusing?
**These entries with ‘complex reporting’ are those where Injuries were classified by type of exam, and could be narrative in nature. Because of the highly detailed nature and formatting of the injury information in comparison to the relatively simple information reported on state-required forms, the process of “translating” those medical notes into something more similar to the rest of the data would be highly time consuming. All rehabilitation records were individually read and manually recorded into a spreadsheet so that the data could be organized; due to the much greater length and depth of Tri-State Bird Rescue and Research injury and exam recordkeeping compared to all other records provided to us, we could not include it with the time constraints we had.**

Line 160 – correct me if I am wrong but this is the first time I have come across “organisational funding” data? What was this used for? Should be included at the end of the introduction where the authors also outline what they did, etc.
**This has been fixed in the manuscript.**

Lines 168-173 – please write in past tense.
**This has been fixed in the manuscript.**

Line 170 – “missingness”?

**This has been changed to “missing data” in the manuscript.**

Lines 167-173 – why did the authors attempt to do this? This seems overkill to me, and introduces an entire suite of biases into the data set. What is wrong with modelling with NAs on subset data sets? Even then, this entire subsection is insufficiently described and requires much more extensive methodological details.
**Multiple imputation is a now-standard method that has been widely used in fields like epidemiology and psychiatry for at least a decade (10.1001/jama.2015.15281; 10.1136/bmj.b2393). It has also been shown to produce more accurate estimates than single imputation (e.g., mean-value imputation) and complete-case analysis (i.e., dropping incomplete rows, as you recommend). As such, we feel that it is more than appropriate in this case.**

**That being said, we agree that more detail is needed in this section. Multiple imputation in R is actually extremely straightforward (a single line of code) so we have simply added an extra sentence justifying its use (with references), the software package that was used, which variables were included, and how the model estimates were pooled afterwards.**

Line 176 – R version needed here. Also spell out abbreviations (not for packages/software, etc.).

**This has been fixed in the manuscript.**

Line 180 – again, correct me if I am wrong but isn’t a logistic model just a binomial model with a logit link function? If so, it might be best to clarify this for the reader.

**Good idea, we have added in parentheses that the logistic model is just a binomial model with a logit link function.**

Line 181 – I am not sure what a “right-censored survival model” is, could the authors please clarify.

**A right-censored survival model is a survival model where the survival times are missing for some individuals, due to death or other factors.** **We have added this clarification to the methods.**

Line 184 – the effects of funding are not included at the end of the introduction (see previous comment).

**This has been corrected.**

Also, this section needs to be revised/clarified. I am assuming that mass, funding, season, etc., were fitted as explanatory variables? I ask only because this has not been sufficiently explained by the authors here.
**Sorry, but we are not sure what clarification is needed. We explicitly list which variables are included as predictor variables in the text (mass, funding, season, and age), and we include Wilkinson notation for the final models that shows all of the predictor variables. In case the confusion is about whether they are fixed or random effects, we have made this more explicit in the test.**

Results
Line 211 – I would suggest to avoid starting your results section with a negative, perhaps “We extracted bird-building collisions data from XX..”.

**This has been fixed in the manuscript.**

Line 220 – too much unnecessary information here, e.g., (PDFs), some of this could be moved to the supplementary material. Actually, I think the entire first paragraph should be moved to the supplementary material, lead the results section with the *current* second paragraph.

**We believe that there is value in reporting how this information was provided, as it provides insights into how the state is managing and processing (or not) the information they are collecting, and what the process of extracting the data from state-provided material entails.**

Line 233 onwards – it may be better to present the % in text and the sample size in parentheses, it would help with the flow of the text.

**This has been fixed in the manuscript.**

Line 236 – 1/3033 is not 0%, replace with <0.1%.

**This has been fixed in the manuscript.**

Line 238 – try to avoid repeating words, e.g., “released”

**This has been fixed in the manuscript.**.

Lines 241-242 – output should stay in results section but the description of the statistical tests used to compare differences should be fully explained in the “statistical analyses” subsection of the methods. Furthermore, where are the test statistics and degrees of freedom? This comment also applies throughout.

**This has been fixed in the manuscript.**.

Line 244 – please write in past tense.

**This has been fixed in the manuscript.**

Line 255 – see comment above.

**We are unsure which comment this is referring to.**

Line 256 – this sentence needs rewording.

**This has been fixed and moved in the manuscript**

Lines 261-268 – this section should be moved up towards the top of the results, perhaps after the sample sizes.

**This has been fixed in the manuscript.**

Fig 1 – all figure and table captions should read independently of the main text, i.e., I should know exactly what the authors’ have done without having to read the main text. At present, this caption is lacking information. This comment applies throughout.

**This has been fixed in the manuscript.**

Lines 307-308 – models do not “find” anything, alternatively they may “show” a pattern. please reword throughout.

**This has been fixed in the manuscript.**

Table 3 – I am confused as to what the 2.5% and 97.5% confidence intervals (?) show here? Aren’t these just lower and upper bounds? In the caption the authors state that 95% Cis were used. There is no mention of any other α-values beside 5% used for the stats. Please clarify.

**These are the upper and lower bounds of the 95% credible intervals from the Bayesian models. We have added a brief mention of this to the tables captions (for both Table 3 and Table 4).**

Discussion
Line 339 – year needed alongside reference.

**This has been fixed in the manuscript.**

Lines 352-353 – I think this should be one of your take-home messages from this study, at the moment, it is not highlighted enough.
**We have reformatted our conclusion, which hopefully makes this point more prominent as a take-home message. However, due to the discrepancy between carcass and rehabilitation-based studies, perhaps the effect of mass on building collision survival requires additional study involving both kinds of datasets before population-level conclusions can be drawn, though this result may have potential patient triaging value to wildlife rehabbers.**

Lines 370-372 – what about increased light levels during summer which may also increase reflections in glass? Has this ever been considered before?

**This is an interesting idea that may be important to examine further in a field-based manuscript.**

Line 381 – scientific name required on first mention, please.

**This has been fixed in the manuscript.**

Lines 387-388 – one limitation of this study is that it was unable to test intraspecific differences in body mass and the effects on survival, which would be interesting.

**Regrettably, this is true. The mass of each patient was not provided in the documents from the state. Future studies with other datasets with more detailed patient information may be able to delve deeper in to this, but it was beyond our capabilities within this study.**

Lines 403-405 – could this not just simply be because there are more adults within a population at any one time than juveniles? So the probability of them colliding with buildings is higher anyway?

**This may be the case, but the cause of collisions is beyond the scope of this manuscript. Because of the frequency of juveniles casualties reported in collision studies, our result finding adults outnumbering juveniles was unexpected.**

Lines 413-415 – I really like the idea of exploring the effects of organisational funding, and feel that the authors could emphasize this more especially in the introduction. This is also another one of the study’s important take-home messages. Move towards the top of the Discussion.

**This is indeed a very interesting part of our study, but there are limitations to our examination of organizational funding. Although we were able to acquire some information, many of these organizations run other programs (usually educational) in addition to their rehabilitation operations, and we do not have enough information on how their budgets are broken down to warrant emphasizing this point any further. We have added addition discussion of the importance of funding to the manuscript.**

Line 423 – this seems like a sensible place to mention large data exercises/databases such as WILD-ONe.

**Mention of WILD-ONe, and WRMD, have been added, thank you for this.**

Lines 433-434 – yes, I absolutely agree.

Lines 436-438 – remove this, it comes across as though the authors are dismissing rehabilitation-focused journals here. Actually, having specialised journals is an excellent resource and a good go-to to find studies specifically on wildlife rehabilitation. I would argue that without them, dissemination would be far more challenging than it is now

**We do not in any way intend to dismiss rehabilitation-focused journals, but the fact of the matter is that unfortunately, the knowledge and experiences of wildlife rehabbers are too often overlooked in academic conservation discussions. We agree with you that the rehab-focused journals and research (and of course their work!) contribute importantly toward conservation aims, but their work should also have a prominent place outside of the wildlife rehabilitation sphere as well.**

Lines 441-455 – references needed throughout this section, there are many studies that mention/discuss biases associated with using rehabilitation centre admissions data. I encourage the authors to ready more widely and with that, cite more widely. Some examples:
<https://www.awrc.org.au/uploads/5/8/6/6/5866843/trocini_surveillance.pdf>
<https://doi.org/10.1002/ece3.8856>

**This has been fixed in the manuscript.**

—-------------------------------------------------------------------------------------------------------------

Reviewer #3: This paper represents an important perspective for interpreting the true mortality impact of window collisions and is a novel use of wildlife rehabilitation data. After some additional analyses, it could be of interest to a broad audience in the conservation community.

Comments on the analysis:
The analyses were sophisticated, I have not done some of the survival analyses but have experience with MCMC glmm and felt that taxonomic traits and facility parameters required better consideration.

The inclusion of taxonomic traits within the model would increase impact and interest if ‘species’ were included within the model, with an additional correction for non-independence for taxonomic relatedness, perhaps in a covariance matrix (see Barrow et al. 2019). The text states that species or families “were not necessary" (lines 184-186), but I feel that estimating these effects is more instructive than mass or age. There are important ecological traits that predispose particular species to collisions and influence the success of rehabilitation, such as typical species temperament, which can influence the level of care that is possible. Estimating species-specific risk on the taxonomy of collision risk, release probability, and duration of care could help inform wildlife rehabilitation guidelines and provide increased information on species-specific susceptibilities, which could also be implemented in mitigation plans. This was also stated in the discussion (lines 397-399) which I agreed with, and it seems that the data and the analysis would support this.
**We acknowledge that incorporating species and accounting for phylogeny would be a useful contribution, but we think it is outside of the scope of this study. First, our statistical models are already quite computationally expensive and difficult to fit, with both the survival modeling and handling of missing data. Second, we have relatively low sample sizes for many of the species we are working with, especially for birds that are released, so it is doubtful that we would be able to accurately estimate species-specific susceptibilities.**

For the facility, I think overall income would be misleading and should be corrected for facility size, the budget for treatment and standardized per animal treated. Many rehabilitation centres have education and outreach programs, so the expenditure on medical treatment is a more important measure. In my experience, facility funding is critical for increasing survivorship - funding is needed to ensure unexpired medications, oxygen chambers, radiographs and medical consumables for treating shock and avoiding sequelae of traumatic brain injuries. As a message in the paper is the important role of rehabilitation centers and how they are underfunded (line 434), it is critical to provide insight into how funding should be allocated. For example, is it better to fund fewer larger facilities with the capability of engaging more stakeholders, such as veterinarians, whose involvement would help improve outcomes and report quality.
**We agree with this point, and we agree that the use of overall income is not ideal. However, this information was all we could obtain based on publicly accessible records. We feel that a more in-depth exploration of this topic would be highly interesting and potentially beneficial, but unfortunately a more in-depth analysis of this was outside of the scope of our study.**

Barrow LN, McNew SM, Mitchell N, Galen SC, Lutz HL, Skeen H, et al. Deeply conserved susceptibility in a multi-host, multi-parasite system. Ecol Lett. 2019;22: 987–998. Pmid:30912262

**We are unsure how to respond to this note and assume it was included in error.**

Comments.

The role of public education in reducing mortality at private residences could be touched upon in the discussion. Very simple window adhesives have been very effective in reducing bird mortality, and it could be beneficial to discuss the products for readers and papers that have tested their efficacy.
Product: <https://www.featherfriendly.com/residential>

**This is an important suggestion and we have added it to the conclusion.**

—-------------------------------------------------------------------------------------------------------------

Reviewer #4: The paper is well written, except for some minor copy editing issues. The language is clear and does not emphasize jargon. I did have some difficulty accessing the data spreadsheets. Please see attached document for detailed comments

This paper addresses a basic but significant gap in the literature – the fate of birds that hit glass but do not die immediately. To grasp the magnitude of mortality from this source, it is essential to learn as much as possible about this group of birds. The authors have worked hard to extract useful information from a very difficult but important data set. The language is clear throughout as is the logic of the analysis. They have done an excellent, and comprehensive review of the relevant literature, including papers that are still in review. There are some relatively minor copy editing issues. I had some difficulty accessing the data spreadsheets.

Specific comments:

L 20 Klem, 1990 and 2024 do suggest die at some rate, so possibly say ‘largely assumed to survive’; similarly line 44 and line 73

**This has been updated in the manuscript.**

L 95/125 – are better data available directly from rehab centers, as opposed to state reports?
**This answer varies on a case-by-case basis. Some rehabbers take highly detailed notes and organize their patient information in databases like Wildlife Rehabilitation Medical Database (WRMD) or the database WILD ONe, while for others the highest-quality information they provide are those government-mandated forms. Even on forms provided to the states, the quality of information (and completion) can vary significantly between facilities and individuals. Even between users of databases, the completeness, detail, and quality of the entries will of course vary to some degree.**

L143 – individual centers may also be a source of variation – are data sources identifiable? (they must be, since funding is a variable)
**This is an important question. The short answer is yes, we have identified many of the data sources, but we do not feel comfortable sharing this data because it would require identifying individual centers and rating them in individual success, which could influence their ability to attract donations or volunteers. Rehabilitation facilities play an important role in protecting wildlife, and we do not want to create more challenges to individual organizations. To answer this question, yes, we managed to identify most individual sources of data, with the exception of organizations whose information was not available with ProPublica or could not be identified with certainty due to redactions or omissions on the forms received from the states (for instance, some information was redacted from PA, and RI did not report to us any organizational or individual information to us at all).**

L150 – what does ‘complex nature of reporting’ mean?
**These entries with ‘complex reporting’ are those where Injuries were classified by type of exam, and could be narrative in nature. Because of the highly detailed nature and formatting of the injury information in comparison to the relatively simple information reported on state-required forms, the process of “translating” those medical notes into something more similar to the rest of the data would be highly time consuming. All rehabilitation records were individually read and manually recorded into a spreadsheet so that the data could be organized; due to the much greater length and depth of Tri-State Bird Rescue and Research injury and exam recordkeeping compared to all other records provided to us, we could not include it with the time constraints we had.**

L156 what was rationale for expecting population density to have an impact?
**Population density was used as a way to estimate the degree of urbanization of a given area. Because urban areas attract higher densities of birds due to artificial light and night, concentrate collisions, and may have different species colliding than rural areas, we considered population density an important factor to consider.**

L160 – why might you expect that organizational funding level would have an impact? **In theory, well-funded organizations would have the capacity to treat more patients than those with less funding, have better access to medical equipment, and provide better quality care to the patients they have. These advantages potentially could translate into higher survival rates for patients that enter facilities with higher funding.**

L215 why not use data from all counties in NY? How many were not selected?
**The New York State Department of Ecological Conservation refused to provide records for the entire state, and instructed us to select specific counties of interest. We selected 9 counties by population and proximity to major cities; 53 remain unselected.**

L230 why was this data not available?
**Although our request for data from the NYSDEC should have contained this facility (Wild Bird Fund), the records were missing. We asked for an explanation as to why, and tried specifically to request this information, but no response was received. We also contacted the facility directly, but they unfortunately refused to supply their records.**

Table 2 – state that families are in order of %release

**We have added this, thank you.**

Seems as though head trauma, concussion and balance issues may not be independent
**This observation may be true but based on the data we received, we cannot be certain. Unfortunately, we are limited by the amount of detail included in the forms, and to try to combine or split into categories would have amounted largely to speculation. Head trauma may be a bruise or cut, but not result in concussion. Balance issues could be due to head trauma, a leg injury, or ingestion of some toxin. Without more information we decided to not change or recategorize how records were received.**

L280 + % released not meaningful to two decimal places

**Corrected, thank you.**

L293 ‘vast majority …. appeared’ – how do you know? Were all examined for parasites?
**Examination for parasites is a typical part of intake physical examinations, and infection of endoparasites usually becomes apparent within the recommended initial 24 hour or more quarantine period of new patients. As part of their normal conduct, wildlife rehabilitators need to take precautions against spreading infection (both microbial and parasitic in nature) among their patients, so identifying such issues are important to rehabilitation environments. Assuming all facilities and individuals in this study were following responsible protocols (which we could not personally verify for all cases, but hopefully is a reasonable assumption), it is reasonable to expect that patients were checked for the presence of parasites.**

L328 ‘no effect …… or … funding’ -- but line 311 indicates no effect of funding, also

**The wording has been modified for clarity in the manuscript.**

L346 It is possible that collisions behavior is quite different at night, with birds not flying directly at glass, but coming down nearby and fluttering towards it – may not be able to generalize from this paper.

**We agree that we cannot determine the difference between night and daytime collisions in this paper, only outcomes as a results of a collision that led to birds being stunned. Differences in the behavior and ecological niche of species and how they affected collisions fell outside of the scope of our study. Additionally, rehabilitation patients were brought in by people who found these birds, but could not know how long they had been there if they did not witness the collision occur. Additionally, although some rehabilitation logs provided admission time of day, we cannot know how soon the bird was brought in after they were found (let alone since the collision), or whether they were left for a rehabber in a medicine box until they could be taken in.**

L365 why would younger birds require protracted care? They have fledged and are migrating
**While yes, all birds that have collided with structures are assumed to have fledged, by no means are all of the younger birds in this dataset migrating. As data was collected in all seasons of the year, especially summer, younger birds that have fledged but may still rely on their parents for extra feeding and are still in a stage of rapid development may require more intensive care. Additionally, while migration behavior fell outside of the scope of our study, juvenile non-migratory birds still hit buildings, and though they may have been in the process of dispersing, they certainly were not in a state of migration.**

L366 you would expect that fractures would take longer to resolve than head trauma; rehabilitators may also be dealing with many more cases in summer; may retain birds rather than release in winter, if weather is bad

**This is an excellent point– we have clarified this in the manuscript.**

L377 Territorial attacks on reflections should not be considered with collisions – they generally do not cause injuries, although the birds may waste significant energy

**While we agree that territorial attacks should not be considered a form of collisions we can not clearly say that birds were not brought to rehabs due to territorial attacks, which can cause bleeding and physical trauma, as well as exhaustion.**

L400-401 Chi square and Fishers? Not mentioned in analysis section

**This has been corrected.**

L414 Well funded orgs may not be able to handle more cases, although they might have better equipment, facilities and access to vet care. How many orgs were involved? Some very poorly funded orgs have very passionate and dedicated rehabilitators.

**Twenty-eight organizations were included in our dataset, and nine individuals either independent or not clearly associated with an organization. Organizations, depending on size, may also spend money on efforts outside of rehabilitation, such as educational outreach. Importantly, we suspect that the dedicated and passionate rehabbers are one of the reasons that funding did not have an effect on release rate; indeed, the nine unaffiliated individuals, assumed to receive no funding whatsoever, collectively had a release rate above 50% (28 released, 18 deceased, 46 total).**

L420-423 duplicated in L423-446

**Corrected, thank you!**

L442 ‘representative’?

**We changed this word to be clearer, thank you!**

L447 starting to be MOTUS data on survival of rehab releases

**Yes, we are eager to see the results of these studies.**

L449 Given that birds may be transported significant distances to rehab facilities, how will more fine grained maps of population density improve the analysis?

**A limitation of our study is that in many cases we could only narrow the incident location down to a town or county based on provided records. If all cases included records of collision sites down to even the zip code, differences in outcomes between collisions happening in urban and rural areas could be better understood.**
